# Two-step clustering-based pipeline for big dynamic functional network connectivity data

**DOI:** 10.3389/fnins.2022.895637

**Published:** 2022-07-25

**Authors:** Mohammad S. E. Sendi, David H. Salat, Robyn L. Miller, Vince D. Calhoun

**Affiliations:** ^1^Wallace H. Coulter Department of Biomedical Engineering, Georgia Institute of Technology and Emory University, Atlanta, GA, United States; ^2^Department of Electrical and Computer Engineering, Georgia Institute of Technology, Atlanta, GA, United States; ^3^Tri-Institutional Center for Translational Research in Neuroimaging and Data Science, Georgia Institute of Technology, Georgia State University, Emory University, Atlanta, GA, United States; ^4^Harvard Medical School, Boston, MA, United States; ^5^Massachusetts General Hospital, Boston, MA, United States; ^6^Department of Computer Science, Georgia State University, Atlanta, GA, United States

**Keywords:** dynamic functional network connectivity, kmeans clustering, human connectome project, big data, reproducibility

## Abstract

**Background:**

Dynamic functional network connectivity (dFNC) estimated from resting-state functional magnetic imaging (rs-fMRI) studies the temporally varying functional integration between brain networks. In a conventional dFNC pipeline, a clustering stage to summarize the connectivity patterns that are transiently but reliably realized over the course of a scanning session. However, identifying the right number of clusters (or states) through a conventional clustering criterion computed by running the algorithm repeatedly over a large range of cluster numbers is time-consuming and requires substantial computational power even for typical dFNC datasets, and the computational demands become prohibitive as datasets become larger and scans longer. Here we developed a new dFNC pipeline based on a two-step clustering approach to analyze large dFNC data without having access to huge computational power.

**Methods:**

In the proposed dFNC pipeline, we implement two-step clustering. In the first step, we randomly use a sub-sample dFNC data and identify several sets of states at different model orders. In the second step, we aggregate all dFNC states estimated from all iterations in the first step and use this to identify the optimum number of clusters using the elbow criteria. Additionally, we use this new reduced dataset and estimate a final set of states by performing a second kmeans clustering on the aggregated dFNC states from the first k-means clustering. To validate the reproducibility of results in the new pipeline, we analyzed four dFNC datasets from the human connectome project (HCP).

**Results:**

We found that both conventional and proposed dFNC pipelines generate similar brain dFNC states across all four sessions with more than 99% similarity. We found that the conventional dFNC pipeline evaluates the clustering order and finds the final dFNC state in 275 min, while this process takes only 11 min for the proposed dFNC pipeline. In other words, the new pipeline is 25 times faster than the traditional method in finding the optimum number of clusters and finding the final dFNC states. We also found that the new method results in better clustering quality than the conventional approach (*p* < 0.001). We show that the results are replicated across four different datasets from HCP.

**Conclusion:**

We developed a new analytic pipeline that facilitates the analysis of large dFNC datasets without having access to a huge computational power source. We validated the reproducibility of the result across multiple datasets.

## Introduction

In recent decades, blood-oxygenation-level-dependent (BOLD) functional magnetic resonance imaging (fMRI) has provided unique information about brain changes associated with various brain disorders (Heeger and Ress, 2002; Poldrack, 2008; Carbó-Carreté et al., 2020). Functional MRI is a non-invasive imaging technique that identifies localized, time-varying alterations in brain metabolism, such as blood flow and deoxygenated hemoglobin levels (Herberholz et al., 2011). These metabolic changes can be induced by a cognitive task (i.e., task-based fMRI) (Cook et al., 2020) or via unregulated brain fluctuations during rest (i.e., resting-state fMRI). Functional connectivity (FC) or its network analog functional network connectivity (FNC) studies the temporal dependence (typically assessed with correlation) between the BOLD fMRI signal from different brain regions (van den Heuvel and Hulshoff Pol, 2010). The FNC approach uses temporal dependence to infer how various brain networks communicate and may play a significant role in understanding how large-scale neuronal communication in the human brain relates to human behavior (Kalinosky et al., 2019; Cook et al., 2020) and how neurodegenerative diseases alter this relationship (Wang et al., 2019; Yan et al., 2019; Hummer et al., 2020; Quevenco et al., 2020; Vega et al., 2020).

Most previous studies assume FNC is static over time and ignore (average out) brain dynamics (Ioannides, 2007). Indeed, FC is highly dynamic, even during the resting state (Sendi et al., 2021c). In recent years, a new line of research called dynamic functional network connectivity (dFNC) has moved beyond studying the strength of connectivity among brain regions and studied the temporal properties of the FNC (Allen et al., 2014). Dynamic FNC has shown promise as a biomarker for schizophrenia (Sendi et al., 2021a,b), Alzheimer’s disease (Sendi et al., 2021c), major depressive disorder (Sendi et al., 2021d), and autism spectrum disorder (Harlalka et al., 2019). It has been shown that dFNC improves the classification of disordered and healthy conditions (Rashid et al., 2016; Saha et al., 2021) and provides more information about neurological and neuropsychiatric disorders pathology than its static counterpart (Menon and Krishnamurthy, 2019).

**FIGURE 1:**
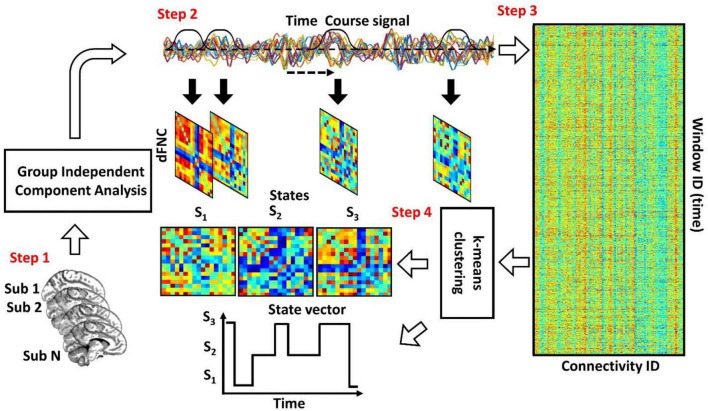
The conventional dFNC pipeline. In Step 1, we estimate the independent components using group independent component analysis. In Step 2, we estimate the dFNC using sliding window. In Step 3, we concatenate all dFNCs across all participants. Then, based on elbow criteria, we estimate the cluster order. In Step 4, we use a standard kmeans clustering approach and calculate the dFNC state for group and state vector for everyone.

Figure 1 shows the conventional analytic pipeline that is used for analyzing dFNC information (Rashid et al., 2016; Sendi et al., 2021a,b,c,d). This pipeline contains four main steps. In the first step, we estimate the intrinsic components for the desired brain regions. Second, we calculate the dFNC using a sliding window. In the third step, we concatenate all dFNCs of all subjects and go through an optimization process to find the clustering order based on the elbow criterion. In the fourth step, we estimate the final dFNC for the whole group and state vector for each individual and calculate the dFNC (or temporal) features for statistical analysis.

In the conventional dFNC pipeline, we mainly use a kmeans clustering approach, even though any clustering approach can be used for clustering dFNC information. This is due to kmeans clustering simplicity in implementation and the ability to scale to a large dataset (Fränti and Sieranoja, 2019). Additionally, it has been shown that kmeans clustering is faster than the other methods such as spectral clustering, density-based spatial clustering of applications with noise or DBSCAN, and mean-shift clustering (McInnes and Healy, 2017). But it is still slow and needs substantial computational power when we work on a sizeable dFNC dataset. On the other hand, recently, the availability of extremely large neuroimaging datasets has made the computational burden of clustering dFNC measurements a significant practical challenge. For example, the UK Biobank dataset released neuroimaging data from more than 40,000 participants (Alfaro-Almagro et al., 2021) and has targeted acquiring data from 100,000 individuals (Alfaro-Almagro et al., 2018). Also, it has been discussed that many neuroimaging analytic pipelines are not scalable for massive data sets, including possibly tens, if not hundreds of thousands of participants (van Horn and Toga, 2014).

There are a few disadvantages of using kmeans clustering in the conventional dFNC pipeline. First, we need to load and feed the entire dFNC information to the kmeans clustering to find the final state. Therefore, we need substantial computational power to analyze the sizeable dFNC information. Second, finding the clustering order or the optimum number of dFNC states takes much time in the conventional dFNC pipeline for the large dFNC information. Therefore, developing a framework that can analyze a large dFNC dataset within a reasonable timeframe in a typical cluster computing environment is needed.

This study introduces a new dFNC pipeline that will mitigate the aforementioned issue of the conventional dFNC pipeline in analyzing large dFNC information. The main principle behind the method is to minimize the need to access a huge computational power while we work with large dFNC information. Therefore instead of loading the entire dFNC data to find the optimum state numbers and final dFNC state, we partially load the data through multiple iterations. In more detail, we locally find the states in each iteration and later aggregate all estimated local dFNC states and estimate the final dFNC state for the entire dataset. Therefore, this new approach does not need a large memory to analyze large dFNC data. We evaluated the reproducibility of the results with both standard and proposed dFNC pipelines across four rs-fMRI sessions of human connectome project (HCP) young adults. Additionally, we compared the time needed to find the optimal cluster number with the proposed pipeline vs. the standard one and showed that our approach is faster than the standard method in finding the cluster order. At the same time, both pipelines generate similar dFNC features after finding the final dFNC states.

## Material and methods

Our analytic pipeline includes rs-fMRI preprocessing, extracting independent components, calculating dFNC, and estimating the cluster order and dFNC states using the proposed dFNC pipeline. The following subsection describes each step in more detail.

### Preprocessing and independent components extraction

**FIGURE 2:**
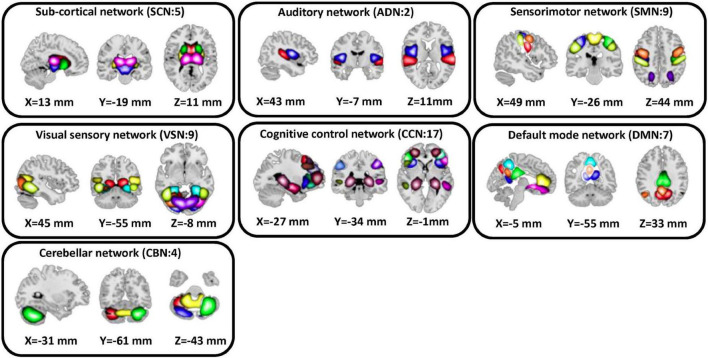
Extracted independent components. Fifty three independent components estimated by NeuroMark pipeline. We put them in seven domains including subcortical network (SCN), auditory network (ADN), sensorimotor network (SMN), visual sensory network (VSN), cognitive control network (CCN), default mode network (DMN), and cerebellar network (CBN).

We used the statistical parametric mapping (SPM12^1^) running in MATLAB2019 to preprocess the fMRI data. The first five dummy scans were removed before preprocessing. Rigid body motion correction was used to account for the participant’s head movement. Then, we used spatial normalization by echo-planar imaging (EPI) template in the standard Montreal Neurological Institute (MNI) space. Finally, a Gaussian kernel was used to smooth the fMRI images using a full width at half maximum (FWHM) of 6 mm. Next, we adapted the Neuromark pipeline to extract intrinsic connectivity networks (ICNs) for each subject (Du et al., 2020). Using this pipeline, we estimated 53 ICNs for each subject and categorized them into seven network domains, including subcortical network (SCN), auditory network (ADN), sensorimotor network (SMN), visual network (VSN), cognitive control network (CCN), the default-mode network (DMN), and cerebellar network (CBN) as shown in Figure 2. The details of the extracted ICNs are provided in (Sendi et al., 2021c).

### Dynamic functional network connectivity estimation

We used a tapered sliding window and estimated the FC within each window using the Pearson correlation, as shown in Eq. 1.


(1) $$R=\frac{\sum_{n=1}^{N}\left(x_{1}-\bar{x_{1}}\right)\,\left(x_{2}-\bar{x_{2}}\right)}{\sqrt{\sum_{n=1}^{N}{\left(x_{1}-\bar{x_{1}}\right)}^{2}}\,\sqrt{\sum_{n=1}^{N}{\left(x_{2}-\bar{x_{2}}\right)}^{2}}}$$

where *x*_1_ and *x*_*2*_ are time-course signals and $\bar{x_{1}}$ and $\bar{x_{2}}$ are the mean of *x*_*1*_ and *x*_*2*_, respectively. It takes values in the interval [−1, 1] and measures the strength of the linear relationship between *x*_*1*_ and *x*_*2*_.

**FIGURE 3:**
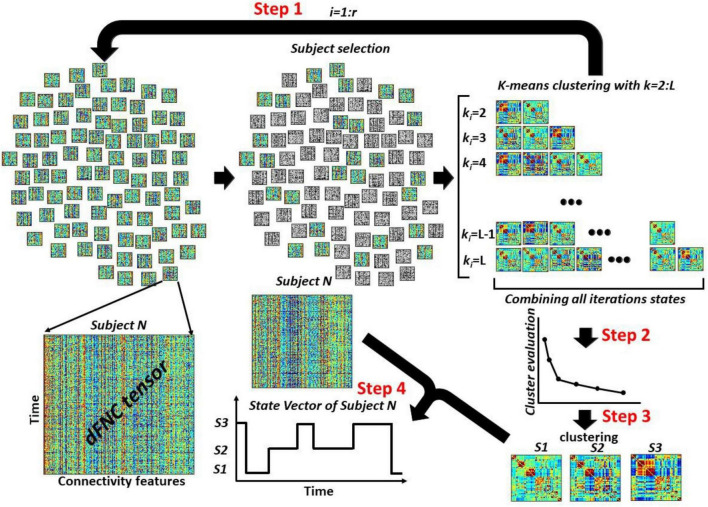
The overview of the proposed dFNC pipeline for dFNC state estimation. In Step 1, we select a subsample of dFNC tensor and then used kmeans clustering with *k*-values from 2 to L and put them into $\left(\frac{L\,\left(L+1\right)}{2}-1\right)$. With *r* iteration, we would have $r\,\left(\frac{L\,\left(L+1\right)}{2}-1\right)$ clusters centroids in total. In Step 2, concatenated all cluster centroids and we use elbow criteria to find the best *k*-values, called K_opt_ hereafter. In Step 3, using another kmeans clustering approach, we estimated the final dFNC states. In Step 4, we used this final states and found the state vector for each subject.

With 53 ICN, the size of each dFNC is 53 × 53, which equals 1,378 distinct connectivity features. Next, we concatenated dFNC estimates of each window for each subject to form a matrix, called dFNC tensor hereafter, with the size of *T × F*, where T denotes the number of windows and F donates the number of connectivity features (Figure 3).

### Proposed dynamic functional network connectivity pipeline

Figure 3 shows the proposed dFNC pipeline for estimating dFNC states of the large dataset. This method includes a few steps. **Step1:** We sub-sample subjects dFNC tensors (*m* subjects from *n* subjects per iteration). Then, we run a standard kmeans clustering on the subsampled data with different values of *k* = 2,3,..,*L*. The k-means algorithm divides *m*×*T* samples *X* of each iteration into *k* disjoint clusters *C*_1_,*C*_2_,…,*C*_*k*_. The cluster centroids μ_*i*_ of *C_i_* minimize the within-cluster sum-of-squares criterion as shown in Eq. 2.


(2) $$\min_{\mu_{1},..,\mu_{k}}(\sum_{j=1}^{k}\sum_{i=1}^{m\,T}\left(||x_{i}-\mu_{j}|{|}^{2}\right)$$

We exhaust all subjects by repeating this process *r* times over disjoint sets of *m* subjects, where *r* is equal to $\frac{n}{m}$. In each iteration, we save all cluster centroids for all values of *k* ∈ [2,*L*]. Therefore, we would have $\frac{L\,\left(L+1\right)}{2}-1$ representative cluster centroids in each iteration. By repeating this process *r* times, we would have $r\,\left(\frac{L\,\left(L+1\right)}{2}-1\right)$ cluster centroids, reducing the data from the whole dFNC. **Step 2:** We concatenate all centroids estimated from all *r* iterations. Next, we use the elbow criteria to find the optimum number of clusters using all $r\,\left(\frac{L\,\left(L+1\right)}{2}-1\right)$ observations. **Step 3:** After finding the optimum number of clusters, called *K*_*opt*_ hereafter, we use another standard k-means clustering to put all $r\,\left(\frac{L\,\left(L+1\right)}{2}-1\right)$ states into K_opt_ cluster, called final states. **Step 4:** Using the final *K*_*opt*_ states, we assign the dFNC of each subject to one of the estimated states and extract the state vector of each participant.

### Dynamic functional network connectivity temporal features estimation

We estimated the occupancy rate (OCR) and the number of transitions between states as the representative dFNC temporal features from the state vector. The OCR represents the proportional amount of time each individual spends in a given state for all HCP datasets through both standard and proposed dFNC pipeline.

### Clustering quality assessment

To assess the clustering quality for each dFNC data, we calculated the distance between the dFNC data and its associated cluster centroid. Then we calculated the distance between each dFNC sample with the other cluster centroids and then summed them up. Then, we calculated the ratio of the latter to the former one for each dFNC instance, called the distance ratio here. Finally, we averaged all distance ratios out for each participant.


(3) $$R_{p}=\frac{1}{T}\,\sum_{i=1}^{T}\frac{d_{i\,\_\,s\,c}}{d_{i\,\_\,c}}$$

*d*_*ic*_ is the distance between each sample to the cluster centroid of the state the sample belongs. Also, *d*_*i_sc*_ is the distance between each sample to other cluster centroids,*R*_*p*_ is the averaged distance ratio for each participant. It is worth mentioning that a higher ratio means better quality in clustering.

### Dataset

To test the proposed method, we used the rs-fMRI and demographic information collected from the 833 young healthy adults (average age: 28.65; range: 22–37 years; female/male: 443/390) from the HCP (Glasser et al., 2016). This dataset is available on the HCP website.^2^ The institutional review board from both Washington University and the University of Minnesota approved the study. The rs-fMRI data were collected on a Siemens Skyra 3T with a 32-channel RF receiver head coil. High resolution T2*-weighted functional images were acquired using a gradient-echo EPI sequence with TE = 33.1 ms, TR = 0.72 s, flip angle = 52°, slice thickness = 2 mm, 72 s slices, and 2 mm isotropic voxel, the field of view: 208 × 180 mm (RO × PE), and duration: 14:33 (min: s). For each participant, four separated rs-fMRI sessions (two sessions per day) were acquired that are called HCP1 (session1, day1), HCP2 (session2, day1), HCP3 (session 1, day2), and HCP4 (session2, day12), hereafter. We used all four sessions to evaluate the reproducibility of the result using the proposed dFNC states estimation method. The dFNC size of HCP1, HCP2, HCP3, and HCP4 is 848,827 × 1,378 (8,542 MB), 732,207 × 1,378 (7403 MB), 747,201 × 1,378 (7,555 MB), and 769,692 × 1,378 (7,742 MB), respectively.

## Results

### Standard and proposed dynamic functional network connectivity pipelines produce similar brain states

**FIGURE 4:**
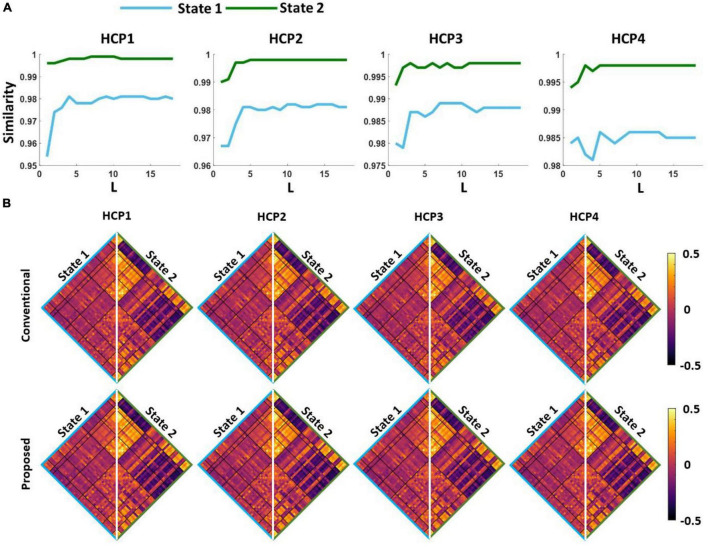
The estimated dFNC states with the proposed and conventional pipeline for all HCP datasets. **(A)** We swept the *L*-value in the first kmeans clustering and calculated the similarity between the estimated states with new and conventional method. For any L > 5, we did not find a significant improvement in the similarity between two clustering methods. **(B)** Both new and conventional pipeline generated similar dFNC states in all four HCP datasets.

The first question we were interested in answering is whether both standard and proposed dFNC pipelines would generate similar dFNC states or not. To test this, we clustered the dFNC data with different *L*-values in the proposed pipeline (as shown in Figure 3). In the new pipeline, we used 3% of the entire dataset in each iteration. Using elbow criteria, we found that the optimal number of clusters is 2 through both conventional and proposed dFNC pipelines. Then, to evaluate the similarity of dFNC states estimated by the proposed pipeline (with different *L*) with the states estimated by conventional kmeans, we used the correlation across the matched states as a similarity metric. The similarity between matched states with varying values of L is shown in Figure 4A for all four HCP datasets. We found that the similarity between the matched states generated by both approaches is more than 99%, with any value L of more than five, and the results were reproduced across four HCP datasets. The estimated states with conventional and proposed dFNC pipelines (*L* = 6) are shown in Figure 4B for all HCP datasets.

### The proposed dynamic functional network connectivity pipeline finds the optimum cluster number faster than the conventional one

**FIGURE 5:**
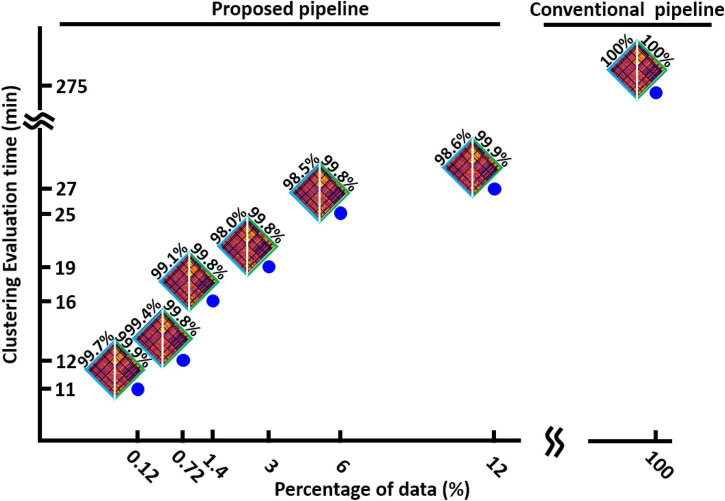
The clustering evaluation time with conventional and proposed method. Reducing the percentage of the data used in each iteration of the first step, reduces the evaluation time. The proposed method is 25 times faster the conventional method. The estimated states and their similarity with states estimated from whole data are shown for each percentage of data.

After finding the minimum reliable value of *L*, we assessed the speed of our method in finding the optimum number of clusters and compared it with the conventional method when it uses the whole dataset. We evaluated the speed of our process with different percentages of data. The result is shown in Figure 5 for HCP1. We found that the new dFNC pipeline is faster when we use a lower percentage of data in each iteration, while the similarity between the matched states estimated with both standard and the proposed pipeline is still more than 98%. Additionally, our proposed method is 25 times faster in funding the cluster order than the traditional method when we use only 0.12% of data (one subject) in each iteration.

### Proposed and conventional dynamic functional network connectivity pipelines generate similar dynamic functional network connectivity features

**FIGURE 6:**
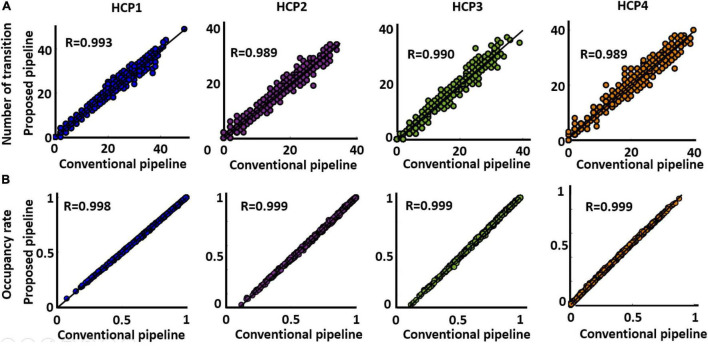
Both standard and the proposed dFNC pipeline generated similar dFNC features replicated across four datasets. **(A)** Estimated number of transitions from both standard and proposed pipeline for all HCP datasets. The similarity between the estimated number of transitions from both methods is more than 0.989. **(B)** Estimated occupancy rate (OCR) from both standard and proposed pipeline for all HCP datasets. The similarity between the OCR from both method is more than 0.989 (*p* < 0.0001, *N* = 833).

The next question is whether both approaches generate similar dFNC features or not. To assess this, we estimated occupancy rate (OCR), the proportional amount of time each participant spends in a specific state, and the number of between-state transition numbers for each participant in both standard and proposed dFNC pipelines. Both features are estimated from the state vector, which shows the state of the brain at a given time (Figure 3, Step 4). Then, to assess the similarity between the two methods in estimated dFNC features, we calculated the correlation between the result of the two methods. The results are shown in Figures 6A,B for OCR and the number of transitions, respectively, for all four HCP datasets. As Figure 6A shows, the correlation between the estimated OCR by conventional and new dFNC pipelines is more than 0.98 (*p* < e^–10^). The result was replicated for all four HCP datasets. Additionally, the number of between-state transitions is significantly similar for both methods, and the result was repeated in all HCP datasets. This piece of evidence shows that our new dFNC pipeline produced similar dFNC features as well as the standard kmeans while our method is faster in finding the clustering order and does not require prohibitive levels of computational power.

### The proposed dynamic functional network connectivity pipeline has better cluster quality than the standard one

**FIGURE 7:**
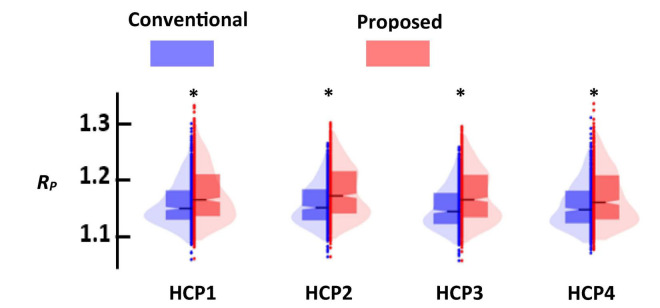
The comparison of the cluster quality between standard (blue) and proposed (red) approach. Each column represents that result of each session. In all comparisons, proposed dFNC pipeline had higher cluster quality (*p* < 0.001, *N = 833*). Asterisk (*) represents a significant different between the clustering quality based of old and proposed.

Figure 7 shows the distance ratio of both standard and new dFNC pipelines for the optimum *k* = 2 values in all four HCP sessions. We used a two-sample *t*-test to compare the distance ratio of the standard vs. the proposed one. We found proposed dFNC pipeline would have better cluster quality than the standard one by having a higher distance ratio (*p* < 0.001, *N* = 833).

## Discussion

In this study, we developed an analytic pipeline to analyze large data dFNC information even without having a sophisticated computational resource. There are a few benefits of using this novel framework. (1) In the conventional dFNC pipeline, we need to load the entire dataset regardless of the clustering approach. Loading the entire dFNC data is computationally demanding and slow when using a large dFNC dataset. Our proposed dFNC pipeline does not require loading the entire dataset. This dramatically reduces the required computational resources and the proposed method can be implemented in a computer with small memory size, (2) we showed our method is 25 times faster than the standard method in finding the cluster order and final dFNC states, (3) we validated the reproducibility of the result across four sessions of rs-fMRI data within a population group; and (4) we demonstrated that our approach generates improved clustering quality compared to the standard approach.

Unlike the conventional dFNC pipeline in which we need to load the entire dataset, our approach loads a portion of the data in each iteration. Therefore, we reduce both the required memory as well as the computational time. Previous studies proposed the mini-batch kmeans that partially loads the data and does not need expensive computational resources (Hicks et al., 2021). But as (Béjar Alonso, 2013) shows, the cluster quality for mini-batch kmeans is reduced compared to standard kmeans clustering, especially when the number of clusters increases. Unlike the mini-batch kmeans approach, our approach reduces the entire clustering process time (Figure 5) and increases the clustering quality compared with standard kmeans (Figure 7). Additionally, we can adapt mini-batch kmeans or other fast clustering approaches to our proposed dFNC pipeline (Viswanath and Suresh Babu, 2009; Choromanska et al., 2013; Pourkamali-Anaraki and Becker, 2017; Chen et al., 2021). In other words, our new approach is a clustering algorithm agnostic pipeline.

Recent approaches for kmeans clustering of big data have focused on identifying the most informative features for the dataset and then running a kmeans on the reduced set. For example, a recent study reduced the dimension of the data set from *p* to *m* (*p* > *m*) by applying a principal component analysis on the entire dataset followed by a kmeans clustering on the projected dataset (Feldman et al., 2013). This method still needs the whole dataset to be loaded, which requires massive computational power. Additionally, since the kmeans is applied to the project space, we do not have an estimation of the cluster centroid in the original space. However, we can transfer the cluster centroid to the original space, but this estimate is inaccurate and yield lower cluster quality than the standard kmeans approach. But, our approach increases the analysis speed without applying any dimensionality reduction approach. Therefore, our method does not lose any information and yields a lower clustering quality.

Our dFNC pipeline is based on the Neuromark pipeline, a fully automated independent component analysis (ICA) framework that uses spatially constrained ICA to estimate components that are flexible to each subject’s data and comparable across individuals (Du et al., 2020). Using the Neuromark pipeline, we calculated the replicated independent components for four HCP sessions. Additionally, we showed that (1) both standard and proposed pipelines generated similar dFNC states in each session of HCP data, and (2) the brain states were replicated across all four sessions using both standard and the proposed dFNC pipeline. The reproducibility of the result across four sessions assessed the robustness of the proposed dFNC pipeline.

There are a few limitations to this study. First, the clustering method in the proposed dFNC pipeline is not limited to kmeans clustering. We can adapt other fast clustering approaches to this pipeline and further improve the computational speed. Second, we did not compare our method’s computational speed and clustering quality with different fast clustering approaches (Viswanath and Suresh Babu, 2009; Choromanska et al., 2013; Pourkamali-Anaraki and Becker, 2017; Chen et al., 2021). However, unlike these fast methods, our approach generated a better quality cluster than the standard kmeans clustering method. A future study is needed to compare the results across multiple clustering approaches. Third, we did not propose an algorithmic approach to set the maximum *L*-value (Figure 3). Finding the optimum *L*-values is done empirically by running the method multiple times to evaluate replicability at different values of *L*. Future study is needed to develop a mathematical approach to finding the optimum *L*-values for each dataset. Additionally, we assumed that the preprocessing, group ICA, and estimating dFNC of the large dataset are already done, requiring considerable computation power for a large dataset. A future study is required to develop a methodology to estimate dFNC information for a large dataset without needing a huge computational power.

## Conclusion

Previous dFNC analytics pipelines use standard kmeans clustering, which is ill-suited for big dFNC data. Here, we developed a new dFNC pipeline that reduced the evaluation time for finding the cluster order while we only loaded a portion of the dataset through several iterations. We validated that our method produces similar brain states and dFNC features as the standard method. Additionally, we evaluated the reproducibility of results across four HCP young adult datasets, which showed the high robustness of the proposed method. There are a few advantages of using the proposed approach over the existing method. (1) In the existing pipeline for analyzing dFNC information, we need to load the entire dataset, which requires a huge computational power. But in the proposed dFNC pipeline, we only need to load a small portion of data in each iteration, and it does not need to have access to a big RAM size to analyze the data. (2) The new existing method can find the optimum number of clusters faster than the existing dFNC pipeline, and (3) we showed that the clustering quality is significantly better than what we can get from the conventional dFNC pipeline.

## Data availability statement

The datasets presented in this study can be found in online repositories. The names of the repository/repositories and accession number(s) can be found below: https://www.humanconnectome.org/study/hcp-young-adult.

## Ethics statement

This study involving human participants was reviewed and approved by the Washington University and the University of Minnesota.

## Author contributions

MS developed the method, analyzed the data, and wrote the manuscript. RM developed the method and provided feedback on the manuscript. DS provided the feedback on the manuscript. VC supervised the study and provided feedback on the manuscript. All authors contributed to the article and approved the submitted version.

## Footnotes

1
https://www.fil.ion.ucl.ac.uk/spm/

2
https://www.humanconnectome.org
